# Observer Agreement on Computed Tomography Perfusion Imaging in Acute Ischemic Stroke

**DOI:** 10.1161/STROKEAHA.119.026238

**Published:** 2019-09-25

**Authors:** Salwa El-Tawil, Grant Mair, Xuya Huang, Eleni Sakka, Jeb Palmer, Ian Ford, Lalit Kalra, Joanna Wardlaw, Keith W. Muir

**Affiliations:** 1 From the Institute of Neuroscience and Psychology, University of Glasgow, Queen Elizabeth University Hospital, Glasgow, Scotland (S.E.-T., K.W.M.); 2 Division of Neuroimaging Sciences, University of Edinburgh, Western General Hospital, United Kingdom (G.M.); 3 Institute of Neuroscience and Psychology (X.H.), University of Glasgow, Scotland; 4 Robertson Centre for Biostatistics (I.F.), University of Glasgow, Scotland; 5 Department of Neuroimaging Sciences, Centre for Clinical Brain Sciences, University of Edinburgh, United Kingdom (E.S., J.P.); 6 Department of Basic and Clinical Neurosciences, Institute of Psychiatry, Psychology and Neurosciences, King’s College London, United Kingdom (L.K.); 7 Division of Neuroimaging Sciences and UK Dementia Research Institute at the University of Edinburgh, United Kingdom (J.W.).; Stroke Centre, Ospedale Sacro Cuore Don Calabria, Negrar, Italy.; Unit of Neuroradiology, NHS & University General Hospital “Santa Maria alle Scotte,” Siena, Italy.; Acute Stroke Unit, Worcestershire Royal Hospital, Worcester, United Kingdom.; Department of Diagnostic Radiology, Danderyd Hospital, Stockholm, Sweden.; UCL St Luc, Brussels, Belgium.; Department of Radiology, Danderyd Hospital, Stockholm, Sweden.; Stroke Unit, Neurology Clinic, Modena, Italy.; Neuroradiology Department, Hospital Geral de Santo António, Porto, Portugal.; Department of Elderly Care, Bradford Royal Infirmary, Bradford, United Kingdom.; University Hospital of North Staffordshire, Stoke-on-Trent, United Kingdom.; National Hospital, London, United Kingdom.; Department of Radiology, Addenbrookes Hospital, Cambridge, United Kingdom.; University Hospital of Copenhagen, Copenhagen, Denmark.; Department of Neuroradiology, Royal Victoria Infirmary, Newcastle upon Tyne, United Kingdom.; Department of Internal Medicine, Oslo University Hospital, Oslo, Norway.; University of Newcastle Upon Tyne, Newcastle, United Kingdom.; Addenbrookes Hospital, Cambridge, United Kingdom.; Kärnsjukhuset Skövde, Sweden.; Department of Neurology, Arsava Hacettepe University, Ankara, Turkey.; Southern General Hospital, Glasgow United Kingdom.; Radiology Department, Darent Valley Hospital, Dartford, United Kingdom.; Department of Neuroradiology, Salford, United Kingdom.; Azienda Ospedaliero-Universitaria “Ospedali Riuniti” di Foggia, Foggia, Italy.; Department of Neuroradiology, Atkinson Morley’s Hospital, London, United Kingdom.; Queen Elizabeth Hospital, Birmingham, United Kingdom.; Institute for Neurological Sciences, Southern General Hospital Glasgow, United Kingdom.; University Hospital Llandough, Cardiff, United Kingdom.; Hospital Geral de Santo António, Porto, Portugal.; Hospital Clinico San Carlos, Madrid, Spain.; Department of Neurology, King’s College Hospital, London, United Kingdom.; Narsjukvardskliniken. Hassleholm, Sweden.; Serviço de Neurologia, Hospital Geral de Santo António, Porto, Portugal.; Universitätsklinikum, Mannheim, Mannheim, Germany.; Poole Hospital, Poole, United Kingdom.; St Mary’s NHS Trust, London, United Kingdom.; St Mary’s NHS Trust, London, United Kingdom.; Hospital Clínico Universitario, Valladolid, Spain.; Norfolk and Norwich University Hospital, Norwich, United Kingdom.; Karolinska Hospital, Stockholm, Sweden.; Institute of Neuroscience, University of Newcastle Upon Tyne, Newcastle, United Kingdom.; University Hospital of North Staffordshire, Stoke-on-Trent, United Kingdom.; Department of Stroke Medicine, Swansea NHS Trust, Swansea, United Kingdom.; Royal Infirmary Of Edinburgh, Edinburgh, United Kingdom.; Neuroradiologisches Klinik, Universität, Dresden, Germany.; Essex Centre for Neurological Sciences, Romford, United Kingdom.; Brighton & Sussex University Hospitals, Brighton, United Kingdom.; William Harvey Hospital, Ashford, United Kingdom.; Addenbrookes Hospital, Cambridge, United Kingdom.; Department of Radiology, Addenbrookes Hospital, Cambridge, United Kingdom.; Leeds General Infirmary, Leeds, United Kingdom.; Karolinska Hospital, Stockholm, Sweden.; Department of Radiology, Queen Elizabeth Hospital, Birmingham, United Kingdom.; Universität Dresden, Dresden, Germany.; Royal Infirmary Of Edinburgh, Edinburgh, United Kingdom.

**Keywords:** brain, cerebral blood flow, computed tomography, patient selection, perfusion

## Abstract

Computed tomography (CT) perfusion (CTP) provides potentially valuable information to guide treatment decisions in acute stroke. Assessment of interobserver reliability of CTP has, however, been limited to small, mostly single center studies. We performed a large, internet-based study to assess observer reliability of CTP interpretation in acute stroke.

**Methods—:**

We selected 24 cases from the IST-3 (Third International Stroke Trial), ATTEST (Alteplase Versus Tenecteplase for Thrombolysis After Ischaemic Stroke), and POSH (Post Stroke Hyperglycaemia) studies to illustrate various perfusion abnormalities. For each case, observers were presented with noncontrast CT, maps of cerebral blood volume, cerebral blood flow, mean transit time, delay time, and thresholded penumbra maps (dichotomized into penumbra and core), together with a short clinical vignette. Observers used a structured questionnaire to record presence of perfusion deficit, its extent compared with ischemic changes on noncontrast CT, and an Alberta Stroke Program Early CT Score for noncontrast CT and CTP. All images were viewed, and responses were collected online. We assessed observer agreement with Krippendorff-α. Intraobserver agreement was assessed by inviting observers who reviewed all scans for a repeat review of 6 scans.

**Results—:**

Fifty seven observers contributed to the study, with 27 observers reviewing all 24 scans and 17 observers contributing repeat readings. Interobserver agreement was good to excellent for all CTP. Agreement was higher for perfusion maps compared with noncontrast CT and was higher for mean transit time, delay time, and penumbra map (Krippendorff-α =0.77, 0.79, and 0.81, respectively) compared with cerebral blood volume and cerebral blood flow (Krippendorff-α =0.69 and 0.62, respectively). Intraobserver agreement was fair to substantial in the majority of readers (Krippendorff-α ranged from 0.29 to 0.80).

**Conclusions—:**

There are high levels of interobserver and intraobserver agreement for the interpretation of CTP in acute stroke, particularly of mean transit time, delay time, and penumbra maps.

## Abstract 2

Supplemental Digital Content is available in the text.

The use of computed tomography (CT) perfusion (CTP) imaging to assess patients with acute ischemic stroke can improve patient selection for revascularisation therapy^1,2^ and may have particular value in selection beyond current time windows. Widespread use of CT perfusion in routine clinical practice is hindered by methodological differences in scan acquisition, scan processing, and interpretation,^3^ as well as inconsistency in perfusion parameters used for patient selection. Previous studies assessing interobserver reliability of CTP have been limited to mostly single center studies with 2 to 4 observers^4–11^ (Table I in the online-only Data Supplement).

In this study, we used an online platform to assess observer agreement on qualitative interpretation of processed CT perfusion maps, using observers of different specialty and experience.

## Methods

The data that support the findings of this study are available from the corresponding author upon reasonable request

We used the Systematic Image Review System 2 platform, provided by the University of Edinburgh, an established method similar to that used in the ACCESS (Acute Cerebral CT Evaluation of Stroke Study) of observer reliability of plain CT in stroke^12^ and to assess >7000 brain scans in the IST-3 (Third International Stroke Trial)^13,14^ and other ongoing trials.

We selected 24 cases from 2 clinical trials: IST-3 (imaging substudy)^15^ and the ATTEST trial (Alteplase Versus Tenecteplase for Thrombolysis After Ischaemic Stroke)^16^ and 1 observational study, the POSH study (Post Stroke Hyperglycaemia).^17^ Patients with ischemic stroke in all of these studies had CTP performed within 6 hours of symptom onset. All 3 studies were approved by relevant research ethics committees, and all participants had given consent for their anonymized scans to be used in further research.

For standardization, all raw perfusion imaging data were postprocessed by one researcher on a commercially available software platform (MiStar, Apollo Medical Imaging Technology, Melbourne, Australia) to produce maps of cerebral blood volume (CBV), cerebral blood flow (CBF), mean transit time (MTT), delay time (DT), and thresholded penumbra maps (PM). PMs dichotomized the lesion into core, defined as tissue with CBF of <40% and relative DT of >2 s compared with homologous tissue in the contralesional hemisphere, and penumbra, defined as tissue with relative DT of >2 s but relative CBF of ≥40% of that in the contralesional hemisphere.^18^ Scans varied in their range of *z*-axis coverage and in the extent of the visible perfusion lesions. All scans covered the level of the basal ganglia and supraganglionic level required for calculation of Alberta Stroke Program Early CT (ASPECT) score.

Clinicians were invited to participate in the study through advertisement at stroke meetings and emails sent through professional organizations’ and trials’ mailing lists. Scan readers’ details were collected at study registration including age, specialization, country and hospital where they worked, years of experience in their specialties, and frequency of reviewing stroke imaging and perfusion imaging. Participants were allowed to review as many scans as they could, in any order. Observers were presented with noncontrast CT (NCCT) and perfusion maps. A short clinical vignette which included age, National Institutes of Health Stroke Scale, and time from onset to scan was presented to observers after scans were fully reported. Participants could alter window settings for the NCCT, but not for the perfusion maps.

A structured questionnaire, adapted from a previously validated ischemic stroke questionnaire,^12,14,19^ asked observers to comment on presence of an acute ischemic lesion on NCCT, its size, location, swelling, presence of a hyper-attenuated artery, background brain changes (old infarct, leukoaraiosis, brain atrophy), and on the presence of any perfusion deficit, its extent compared with ischemic changes on NCCT reflecting a previously used approach for assessing mismatch of perfusion lesions relative to the visible CT (or magnetic resonance imaging) lesion (no perfusion deficit <20% difference, equal volumes, >20% difference compared with the NCCT hypodensity), and the ASPECT score for hypodensity on NCCT and CTP maps. ASPECT score is a well-established semiquantitative scoring system for assessing the anatomic extent of acute ischemic changes in anterior circulation stroke^20^ (http://www.aspectsinstroke.com) initially developed for NCCT but adapted for analysis of other imaging modalities including CTP.^21^

At the start of the study, the questionnaire only allowed the observer to calculate an ASPECT score if acute ischemic changes were reported in the middle cerebral artery territory, but this restriction was later removed. Observers were also allowed to indicate if an ASPECT score could not be calculated for perfusion maps when the relevant region was not included. The total ASPECT score, ranging from 0 to 10 was used for analyses.

To assess intraobserver agreement, observers who completed all scans were invited to perform repeat review of 6 scans blind to their initial scoring, including 1 with no perfusion deficit, 2 with small to moderate perfusion deficit, and 3 with large perfusion deficit.

Statistical analysis was performed using IBM SPPS software, version 21. Descriptive statistics were used for scan and observer baseline characteristics and χ^2^ tests to compare categorical data between groups. Krippendorff α,^22^ was used to assess inter and intraobserver agreement, using an appropriate macro for SPSS.^23^ Possible Krippendorff-α values range from −1.0 to +1.0, where +1.0 equates to perfect agreement, 0.0 means no agreement, and −1.0 implies perfect disagreement. Levels of agreement were interpreted as follows: 0 to 0.2 =slight, 0.21 to 0.4 =fair, 0.41 to 0.6 =moderate, 0.61 to 0.8 =substantial, 0.81 to 1.00 =almost perfect agreement.^24^ Analysis was performed for all observers and repeated for readers who reported all scans and for different reader and scan subgroups.

## Results

**Figure 1.**
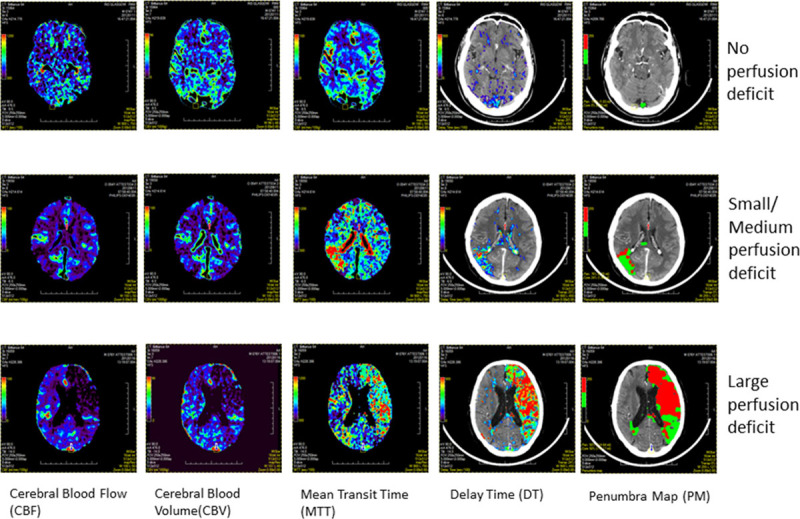
Examples of scans used in the study. Scans were selected to show variable sizes of perfusion deficit as seen on penumbra map.

The clinical and radiological features of the 24 cases used in the study are summarized in Table 1.Scans were classified into 3 groups based on visual assessment of the perfusion deficit on PM: no perfusion deficit (4 scans), mild to moderate perfusion deficit (11 scans), and large perfusion deficit (9 scans). Examples are shown in Figure 1. Perfusion scans varied in their *z* coverage and slice thickness. Most scans (13/24) covered a 4 cm slab, acquired as 8×5 mm slices.

**Table 1.**
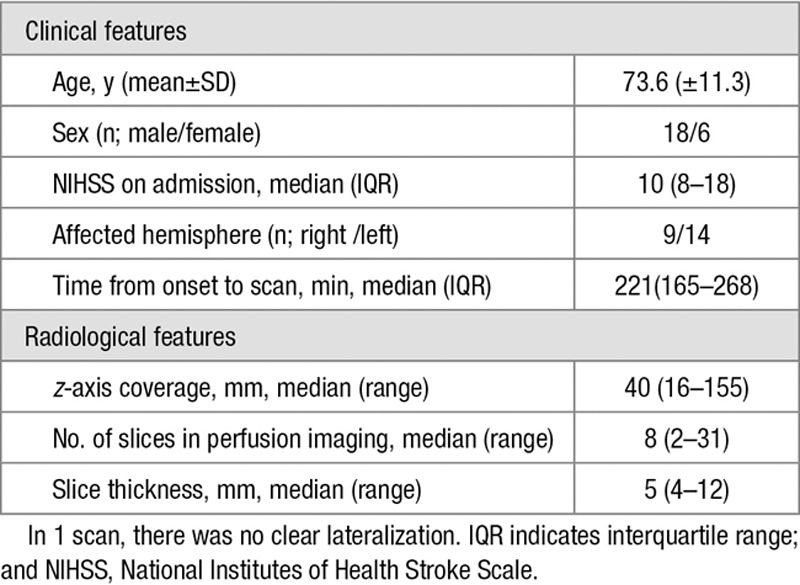
Clinical and Radiological Features of Cases Used

**Figure 2.**
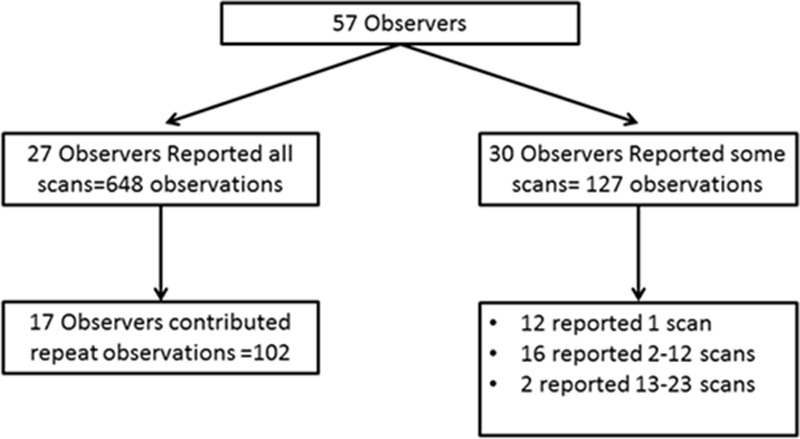
Number of scan reviews generated.

Fifty-seven observers participated in the study (Table 2), of whom 27 (47%) reviewed all scans, and 17 out of 27 (63 %) contributed repeat readings to determine intraobserver agreement (Figure 2). Reviewers were from 10 countries, with most (36/57, 63%) working in the United Kingdom. There was no difference in experience, distribution of specialties, or frequency of viewing stroke scans between the observers who completed all scans and those who did not (Table II in the online-only Data Supplement). The median number of scans reviewed per observer was 19 (interquartile range, 2–24; Figure I in the online-only Data Supplement). Observers tended to read scans in the order in which they were presented, with 56 out of 57(98%) of observers reporting scan 1, and 24 out of 57(42%) of observers reporting scan 24.

**Table 2.**
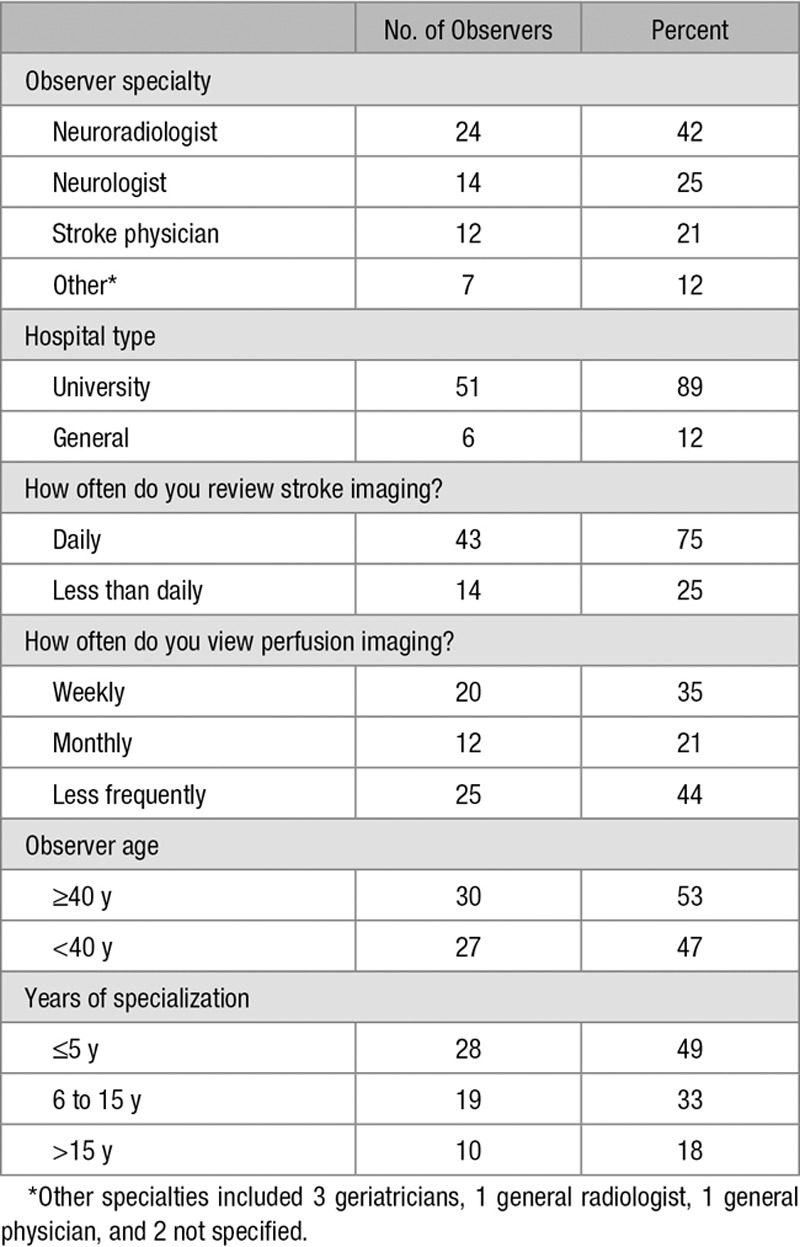
Different Observer Characteristics

**Figure 3.**
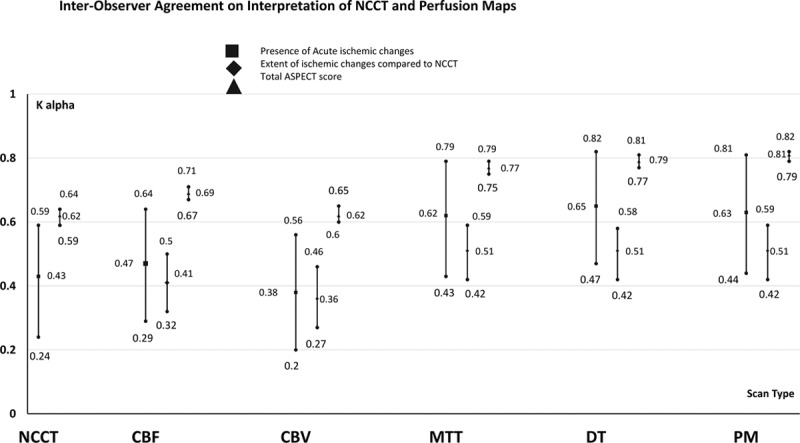
Krippendorff α values and 95% CIs for interobserver agreement on interpretation of noncontrast computed tomography (NCCT) and perfusion maps (PM). ASPECT indicates Alberta Stroke Program Early CT; CBF, cerebral blood flow; CBV, cerebral blood volume; DT, delay time; and MTT, mean transient time.

Interobserver agreement was fair to substantial for recognition of acute lesion on NCCT and on the different perfusion maps and fair to moderate for the extent of CTP-NCCT mismatch (Table III in the online-only Data Supplement) with higher agreement for MTT and DT and PMs. Agreement on total ASPECT score was substantial to almost perfect, with agreement for MTT, DT, and PM higher than agreement for NCCT, CBV, and CBF (Figure 3). Agreement was slightly higher for the group that reviewed all scans compared with those who reviewed some scans only (Figures II, III, and IV in the online-only Data Supplement). Agreement varied among different observer and scan subtypes with the highest agreement among neuroradiologists (Tables IV through XV in the online-only Data Supplement).

**Figure 4.**
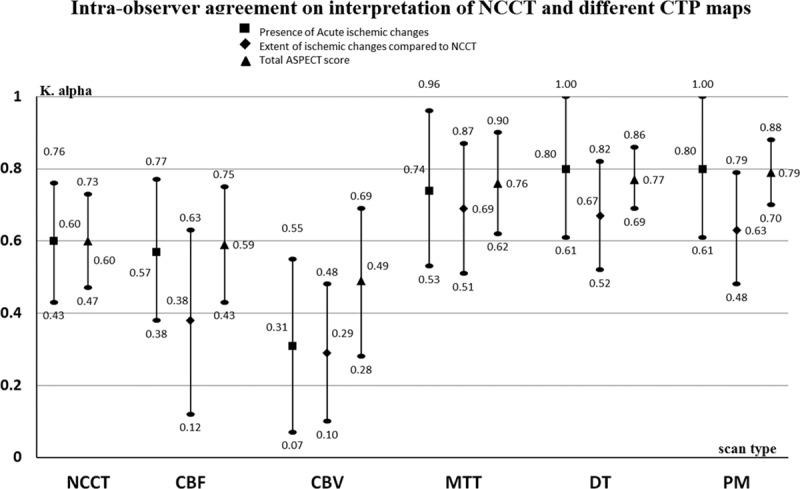
Mean Krippendorff α and 95% CIs of the mean for intraobserver agreement on interpretation of noncontrast computed tomography (NCCT) and perfusion maps (PM). Intraobserver agreement for total Alberta Stroke Program Early CT (ASPECT) score for different sequences. CBF indicates cerebral blood flow; CBV, cerebral blood volume; CTP, computed tomography perfusion; DT, delay time; and MTT, mean transient time.

Seventeen observers contributed to the intraobserver agreement part of the study (results shown in Figure 4). There were no differences in observer characteristics between those who did and did not undertake repeat readings (Table II in the online-only Data Supplement). The time between finishing the first set of readings and starting the repeat reading ranged from 36 to 314 days (median=233 days.). The shortest duration between finishing the first reading and starting repeat reading was 1 month. Levels of agreement varied widely between observer subgroups from slight to almost perfect (Tables XVI through XXVII in the online-only Data Supplement), with highest agreement for time-based parameters.

## Discussion

The role of CTP in patients with acute stroke presenting within treatment windows of 4.5 hours for intravenous thrombolysis or 6 hours for endovascular thrombectomy remains uncertain, with some centers advocating routine use,^25^ whereas others do not undertake CTP routinely due to concerns, such as delayed door to needle time or physician unfamiliarity.^26,27^ Inconsistency in analysis software and proposed analysis parameters is likely to contribute to uncertainty and delay. Our study showed moderate to near-perfect interobserver and intraobserver agreement for time-based CTP parameters (DT and MTT) across observers with a wide range of experience and backgrounds.

Our results are consistent with previous studies that included few observers and fewer scans^4–11^ and extend these previous reports by using a wider spectrum of observers and a larger number of scans, including repeat assessment to establish intraobserver agreement. Differences in the statistical measures used mean that direct comparisons with previous studies are difficult, but the main findings are in agreement, namely, that interobserver agreement is higher for perfusion parameters compared with NCCT, for time-based perfusion parameters (MTT, DT) and for penumbra/core dichotomized maps compared with CBV and CBF. More extensive brain coverage improved interobserver agreement. These trends were stable across all scan and reader subgroups. We also found higher agreement for neuroradiologists versus other specialties, although this was not explained by frequency of interpreting stroke or perfusion scans or years in speciality. Agreement was low in scans with limited *z*-axis coverage, but the size of the perfusion lesion had no effect.

The visual comparison of the perfusion lesion relative to the NCCT lesion size is analogous to the mismatch approach initially described for magnetic resonance imaging, with the hypoattenuation of brain on NCCT corresponding to ischemic core of largely irreversible damaged tissue^28^ and more extensive hypoperfused tissue defined by prolonged MTT or DT corresponding to the potentially reversible penumbra. Because the conspicuity of acute ischemic tissue on NCCT is poor compared with diffusion-weighted magnetic resonance imaging, it is unsurprising that the agreement was lower for mismatch than for ASPECTS, which only depends on the perfusion component. Reduced CBF or CBV on CTP correlate more closely with the diffusion-weighted imaging lesion, but the discrimination of normal from reduced CBV or CBF is more difficult compared with time-based parameters. In general, MTT and DT are more uniform across gray and white matter, and between brain regions, than CBF and CBV which differ more between gray and white matter, and between brain regions, although all perfusion parameters are abnormal in old ischemic lesions and regions of leukoaraiosis.^29^ CBV additionally has a narrow range of normal values. Interobserver and intraobserver agreement for CBF and CBV was similar to that for NCCT. Observer agreement for ASPECT scores applied to perfusion maps showed better agreement for time-based CTP and PMs compared with NCCT, CBF, or CBV, with agreement levels unaffected by the size of the ischemic lesion. This suggests that the visual interpretation of ischemic core on CT-based imaging is more consistent when readers use time-based perfusion parameters and a standardized scoring tool.

In previous studies, the volume of ischemic core tissue was weakly associated with higher risk of adverse outcomes in patients treated with reperfusion therapies, including for infarct swelling and symptomatic intracerebral haemorrhage^30,31^; in several recent trials, a large core constituted an exclusion criterion, although an interaction of core volume and treatment effect could not be demonstrated.^15,32^

The r esults of this study should be interpreted in view of its limitations. The observers were volunteers, presumably having some interest in CT perfusion imaging, and agreement may be different in the wider population of doctors routinely dealing with stroke imaging. The online platform experience deviates from real-life experience in several important aspects; all parameters are available to the observer simultaneously, so that interpretation of NCCT can be influenced by findings in CTP, as appears to have been the case here, and the time pressure related to reviewing a scan to make a treatment decision is not a factor. To reduce observer bias in the study through knowing stroke severity, the clinical information was introduced after the scans were reviewed to test readers’ ability to detect scan findings without influence of clinical findings, but this is the reverse of clinical practice. The range of abnormalities in the scans we selected was limited to patients with mainly middle cerebral artery territory strokes. Finally, these results are limited to visual interpretation of perfusion maps produced by a single software and may not represent agreement with other software or means of presenting perfusion data.

## Summary

Observer agreement on interpretation of CTP perfusion was fair to substantial, with better agreement for time-based sequences (MTT, DT) and threshold-based core/PMs than with CBF, CBV, or NCCT hypoattenuation. Agreement was higher when using the ASPECT score, compared with estimating the size of the perfusion lesion relative to the NCCT lesion, or to the presence versus absence of the perfusion lesion. Intraobserver agreement was also better with time-based maps, but varied significantly among individuals, showing opportunity for improvement.

## Appendix

### Study Collaborators

Alessandro Adami, Stroke Centre, Ospedale Sacro Cuore Don Calabria, Negrar, Italy. Alfonso Cerase, Unit of Neuroradiology, NHS & University General Hospital “Santa Maria alle Scotte,” Siena, Italy. Ana Garcia, Acute Stroke Unit, Worcestershire Royal Hospital, Worcester, United Kingdom. Anders von Heijne, Department of Diagnostic Radiology, Danderyd Hospital, Stockholm, Sweden. Andre Peeters, UCL St Luc, Brussels, Belgium. Anders von Heijne, Department of Radiology, Danderyd Hospital, Stockholm, Sweden. Andrea Zini, Stroke Unit, Neurology Clinic, Modena, Italy. Angelo Carneiro, Neuroradiology Department, Hospital Geral de Santo António, Porto, Portugal. Chris Patterson, Department of Elderly Care, Bradford Royal Infirmary, Bradford, United Kingdom. Christine Roffe, University Hospital of North Staffordshire, Stoke-on-Trent, United Kingdom. Daniel Freedman, National Hospital, London, United Kingdom. Daniel Scoffings, Department of Radiology, Addenbrookes Hospital, Cambridge, United Kingdom. Derk W Krieger, University Hospital of Copenhagen, Copenhagen, Denmark. Dipayan Mitra, Department of Neuroradiology, Royal Victoria Infirmary, Newcastle upon Tyne, United Kingdom. Eivind Berge, Department of Internal Medicine, Oslo University Hospital, Oslo, Norway. Elena Adela Cora, University of Newcastle Upon Tyne, Newcastle, United Kingdom. Eoin O’Brien, Addenbrookes Hospital, Cambridge, United Kingdom. Eric Bertholds, Kärnsjukhuset Skövde, Sweden. Ethem Murat, Department of Neurology, Arsava Hacettepe University, Ankara, Turkey. Fiona Moreton, Southern General Hospital, Glasgow United Kingdom. Garryck Tan, Radiology Department, Darent Valley Hospital, Dartford, United Kingdom. Gillian Potter, Department of Neuroradiology, Salford, United Kingdom. Giuseppe Rinaldi, Azienda Ospedaliero-Universitaria “Ospedali Riuniti” di Foggia, Foggia, Italy. Jeremy Madigan, Department of Neuroradiology, Atkinson Morley’s Hospital, London, United Kingdom. Joe Leyon, Queen Elizabeth Hospital, Birmingham, United Kingdom. Johann Du Plessis, Institute for Neurological Sciences, Southern General Hospital Glasgow, United Kingdom. Jonathan Hewitt, University Hospital Llandough, Cardiff, United Kingdom. José Eduardo Alves, Hospital Geral de Santo António, Porto, Portugal. Jose Egido, Hospital Clinico San Carlos, Madrid, Spain. Laszlo Sztriha, Department of Neurology, King’s College Hospital, London, United Kingdom. Magnus Esbjoernsson, Narsjukvardskliniken. Hassleholm, Sweden. Manuel Correia, Serviço de Neurologia, Hospital Geral de Santo António, Porto, Portugal. Martin Griebe, Universitätsklinikum, Mannheim, Mannheim, Germany. Michelle Dharmasiri, Poole Hospital, Poole, United Kingdom. Olga Kirmi, St Mary’s NHS Trust, London, United Kingdom. Olivia Geraghty, St Mary’s NHS Trust, London, United Kingdom. Pablo García-Bermejo, Hospital Clínico Universitario, Valladolid, Spain. Patrick Sutton, Norfolk and Norwich University Hospital, Norwich, United Kingdom. Pervinder Bhogal, Karolinska Hospital, Stockholm, Sweden. Philip White, Institute of Neuroscience, University of Newcastle Upon Tyne, Newcastle, United Kingdom. Phillip Ferdinand, University Hospital of North Staffordshire, Stoke-on-Trent, United Kingdom. Qazi Anjum, Department of Stroke Medicine, Swansea NHS Trust, Swansea, United Kingdom. Robin Sellar, Royal Infirmary Of Edinburgh, Edinburgh, United Kingdom. Rüdiger von Kummer, Neuroradiologisches Klinik, Universität, Dresden, Germany. Sreeman Andole, Essex Centre for Neurological Sciences, Romford, United Kingdom. Sriram Vundavalli, Brighton & Sussex University Hospitals, Brighton, United Kingdom. Thomas Webb, William Harvey Hospital, Ashford, United Kingdom. Tilak Das, Addenbrookes Hospital, Cambridge, United Kingdom. Tomasz Matys, Department of Radiology, Addenbrookes Hospital, Cambridge, United Kingdom. Tony Goddard, Leeds General Infirmary, Leeds, United Kingdom. Vamsi Gontu, Karolinska Hospital, Stockholm, Sweden. Vijay Sawlani, Department of Radiology, Queen Elizabeth Hospital, Birmingham, United Kingdom. Volker Puetz, Universität Dresden, Dresden, Germany. Will Whiteley, Royal Infirmary Of Edinburgh, Edinburgh, United Kingdom.

## Sources of Funding

This study was funded by the Efficacy and Mechanism Evaluation (EME) Programme (Grant EME 11/100/78), a partnership of the UK Medical Research Council and National Institute for Health Research. The views expressed in this publication are those of the authors and not necessarily those of the Medical Research Council (MRC), National Institute for Health Research or the UK Department of Health and Social Care; the IST-3 (IST-3 (Third International Stroke Trial)) was funded by the UK MRC (G0400069) and the UK Stroke Association among many sources, and the IST-3 perfusion substudy by Efficacy and Mechanism Evaluation programme (09-800-15) and support from many national funding agencies.^33^ ATTEST (Alteplase Versus Tenecteplase for Thrombolysis After Ischaemic Stroke) and the POSH (Post Stroke Hyperglycaemia) studies were funded by the Stroke Association (TSA 2010/04 and TSA 2006/03). IST-3: Drug and placebo for the 300 patients in the double-blind component of the start-up phase of IST-3 were supplied by Boehringer Ingelheim GMBh. Dr Muir received personal fees from Boehringer Ingelheim, Bayer, and Daiichi Sankyo outside the submitted work, and nonfinancial research support from Boehringer Ingelheim for the ATTEST-2 trial.

## Disclosures

None.

## Supplementary Material
