# Supplementary material for: Observer Agreement on Computed Tomography Perfusion Imaging in Acute Ischemic Stroke
Source: Stroke. 2019 Sep 25;50(11):3108–14. doi: 10.1161/STROKEAHA.119.026238 (PMC6824508; doi:10.1161/STROKEAHA.119.026238)
Supplement: Supplementary file 1 [file str-50-3108-s001.pdf]

Supplementary material

Index

- Supplementary table I: Summary of previous studies looking at inter-observer agreement of ASPECT score in different CTP maps.
- Supplementary table II: Observer characteristics
- Supplementary figure I: Number of readings per scan
- Supplementary table III: Observer agreement on relation between ischemic changes on NCCT and different perfusion sequences for all observers
- Supplementary figure II: Observer agreement on presence of acute ischemic changes in NCCT and perfusion maps in for observers reviewing all or some scans
- Supplementary figure III: Observer agreement on extent of acute ischemic changes in NCCT compared to perfusion maps for observers reviewing all or some scans
- Supplementary figure IV: Observer agreement on total ASPECT score in NCCT and perfusion maps for observers reviewing all or some scans
- Supplementary table IV: Observer agreement on presence of acute ischemic changes in different observer specialities
- Supplementary table V: Observer agreement on presence of acute ischemic changes in observers with different experience in their specialities
- Supplementary table VI: Observer agreement on presence of acute ischemic changes in observers with different experience with stroke imaging
- Supplementary table VII: Observer agreement on presence of acute ischemic changes in observers with different experience with perfusion imaging
- Supplementary table VIII: Observer agreement on presence of acute ischemic changes in different scan subtypes
- Supplementary table IX: Observer agreement on extent of acute ischemic changes in NCCT compared to perfusion in different observer subgroups
- Supplementary table X: Observer agreement on extent of acute ischemic changes in NCCT compared to perfusion in different scan subgroups
- Supplementary table XI: Observer agreement on total ASPECT score in observers of different specialities
- Supplementary table XII: Observer agreement on total ASPECT score in observers with different experience in their specialities
- Supplementary table XIII: Observer agreement on total ASPECT score in observers with different experience with stroke imaging
- Supplementary table XIV: Observer agreement on total ASPECT score in observers with different experience with perfusion imaging
- Supplementary table XV: Observer agreement on total ASPECT score in different scan subtypes
- Supplementary table XVI: Intra-observer agreement on presence of acute ischemic changes in different observer specialities
- Supplementary table XVII: Intra-observer agreement on presence of acute ischemic changes in observers with different experience in their specialities
- Supplementary table XVIII: Intra-observer agreement on presence of acute ischemic changes in observers with different experience with stroke imaging

- Supplementary table XIX: Intra-observer agreement on presence of acute ischemic changes in observers with different experience with perfusion imaging
- Supplementary table XX: Intra-observer agreement on extent of acute ischemic changes in NCCT compared to different perfusion maps in observers of different specialities
- Supplementary table XXI: Intra-observer agreement on extent of acute ischemic changes in NCCT compared to different perfusion maps in observers with different levels of experience in their specialities
- Supplementary table XXII: Intra-observer agreement on extent of acute ischemic changes in NCCT compared to different perfusion maps in observers with different experience with stroke imaging
- Supplementary table XXIII: Intra-observer agreement on extent of acute ischemic changes in NCCT compared to different perfusion maps in observers with different experience with perfusion imaging
- Supplementary table XXIV: Intra-observer agreement on total ASPECT score in NCCT compared to different perfusion maps in observers of different specialities
- Supplementary table XXV: Intra-observer agreement on total ASPECT score in NCCT compared to different perfusion maps in observers with different levels of experience in their respective specialities
- Supplementary table XXVI: Intra-observer agreement on total ASPECT score in NCCT compared to different perfusion maps in observers with different experience with stroke imaging
- Supplementary table XXVII: Intra-observer agreement on total ASPECT score in NCCT compared to different perfusion maps in observers with different experience with perfusion imaging.
- References

No	Ref	Scan no	Readers	Statistical test	NCCT (CI)	CBV (CI)	CBF (CI)	MTT (CI)	DT (CI)	Other sequences tested
1	Aviv et al, 2007[1]	36	2 neuro-radiologists	ICC	0.65 (0.49-0.78)	0.69 (0.54-0.81)	0.82 (0.72-0.89)	0.81 (0.70-0.89)	NA	NA
2	Popiela et al, 2008[2]	34	2 pairs	Kappa	0.27	0.57	0.46	NA	NA	TTP
3	Finlayson et al, 2013[3]	180	2 neuro-radiologists<br>2 neurologists	ICC For ASPECT	0.83 (0.79-0.87)	0.90 (0.88-0.92)	NA	NA	NA	CTA-SA
4	Van Seeters et al, 2013[4]	105	2 neuro-radiologists	Kappa for any ischemic changes	0.54	0.80	NA	0.90	NA	PM<br>IM<br>CTA-SA
			2 radiology residents	ICC for any ischemic changes	0.54	0.81	NA	0.92	NA	PM<br>IM<br>CTA-SA
5	Psychogios et al, 2013[5]	51	2 experience neuro radiologists	Weighted kappa for dichotomised ASPECT	0.71 (0.56-0.85)	0.81 (0.69-0.93)	0.91 (0.82-0.90)	NA	NA	NA
6	Khaw et al, 2016[6]	22	2 neuro-radiologist, on stroke neurologists	ICC for ASPECT score	NA	0.58 (0.25-0.79)	0.56 (0.09-0.82)	NA	NA	TTP
7	Shankar et al ,2016[7]	32	1 neuro-radiologist<br>1stroke physician<br>1-radiology trainee	Kappa for presence of abnormalities	0.25 (0.07-0.44)	0.75 (0.57-0.93)	0.79 (0.61-0.97)	NA	NA	TTP
				ICC for ASPECT scores	0.39 (0.18-0.57)	0.89 (0.81-0.94)	0.90 (0.82-0.95)	NA	NA	TTP
8	Naylor et al, 2017[8]	227	2 stroke research fellows	Weighted kappa Repeated for 4 different time epochs	0.76-0.93	0.94-0.95	0.92-0.95	NA	NA	T max

Supplementary table I: Summary of previous studies looking at inter-observer agreement of ASPECT score in different CTP maps. NCCT= non contrast CT, CBF= cerebral blood flow, CBV= cerebral blood volume, MTT= mean transit time, DT, delay time, TTP= time to peak, CTA-SA CT angiography source images, Tmax= . IM=

		First Reading			Second Reading		
		Reviewed all scans (27 total) n(%)	Reviewed some scans (30 total) n (%)	P	Contributed to second reading (17 total); n (%)	Did not Contribute to second reading (10 total);n (%)	P
Observer Speciality	Neuro-radiologist	12(44)	12(40)	0.25	7(41)	5(50)	0.98
	Neurologist	9(33)	5(16)		6(35)	3(30)	
	Stroke physician	3(11)	9(30)		2(12)	1(10)	
	Other	3(11)	4(13)		2(12)	1(10)	
Years of specialisation	0 to 5 years	11(41)	17(57)	0.45	8(47)	3(30)	0.15
	6 to 15 years	10(37)	9(30)		4(24)	6(60)	
	>15 years	6(22)	4(13)		5(29)	1(10)	
How often do you review stroke imaging?	Daily	22(81)	21(70)	0.32	15(88)	7(70)	0.24
	Less than daily	5(19)	9(30)		2(12)	3(30)	
How often do you view perfusion imaging?	Weekly	12(44)	8(27)	0.37	6(35)	6(60)	0.43
	Monthly	5(19)	7(23)		4(24)	1(10)	
	Less frequently	10(37)	15(50)		7(41)	3(30)	
Observer Age	40 years or more	14(52)	16(53)	0.91	9(53)	5(50)	0.88
	Less than 40 years	13(48)	14(47)		8(47)	5(50)	
Hospital type	University/Teaching	24(89)	27(90)	0.89	15(88)	9(90)	0.89
	General/Regional	3(11)	3(10)		2(12)	1(10)	
Country	United Kingdom	12(44)	24(80)	0.01*	6(35)	6(60)	0.26
	Other country	15(56)	6(20)		11(65)	4(40)	

*Supplementary Table II: observer characteristics. ‘Other’ specialities included 3 geriatricians, 1 general radiologist, 1 general physician and 2 not specified. ‘Other’ countries included Sweden (5), Italy (4), Germany (3), Portugal (3), Spain (2), Belgium (1), Denmark (1), Norway (1) and Turkey (1). With the exception of country of residence, there were no statistically significant differences between observers who reviewed all or some scans, and no differences between those who contributed or did not contribute to second reading. *statistically significant*

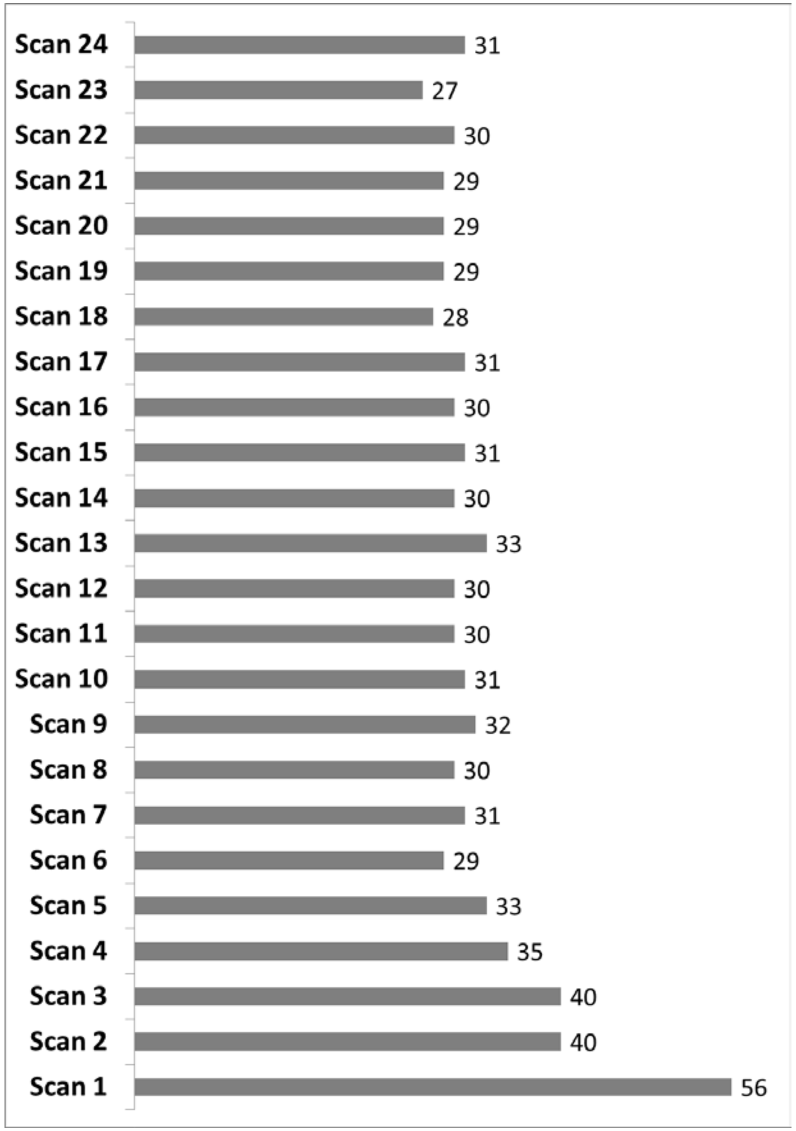

Supplementary figure I: number of readings per scan

	No perfusion abnormality (%)	Perfusion abnormality <20% of NCCT lesion (%)	Perfusion abnormality same as NCCT (%)	Perfusion abnormality >20% of NCCT lesion (%)	Krippendorff α (CI)
**CBV**	355(46)	93(12)	166(21)	161(21)	0.36(0.27-0.46)
**CBF**	267(35)	62(8)	184(25)	252(32)	0.41(0.32-0.50)
**MTT**	201(26)	27(4)	99(12)	448(58)	0.51(0.42-0.59)
**DT**	197(25)	36(5)	114(15)	428(55)	0.50(0.42-0.58)
**PM**	195(25)	38(5)	107(14)	435(56)	0.51(0.42-0.59)

Supplementary table III: Krippendorff alpha values and 95% confidence intervals for observer agreement on relation between ischemic changes on NCCT and different perfusion sequences (CI= confidence interval,NCCT= non contrast computed topography, CBF= cerebral blood flow, CBV= cerebral blood volume, MTT= mean transient time, DT= delay time, PM= penumbra map)

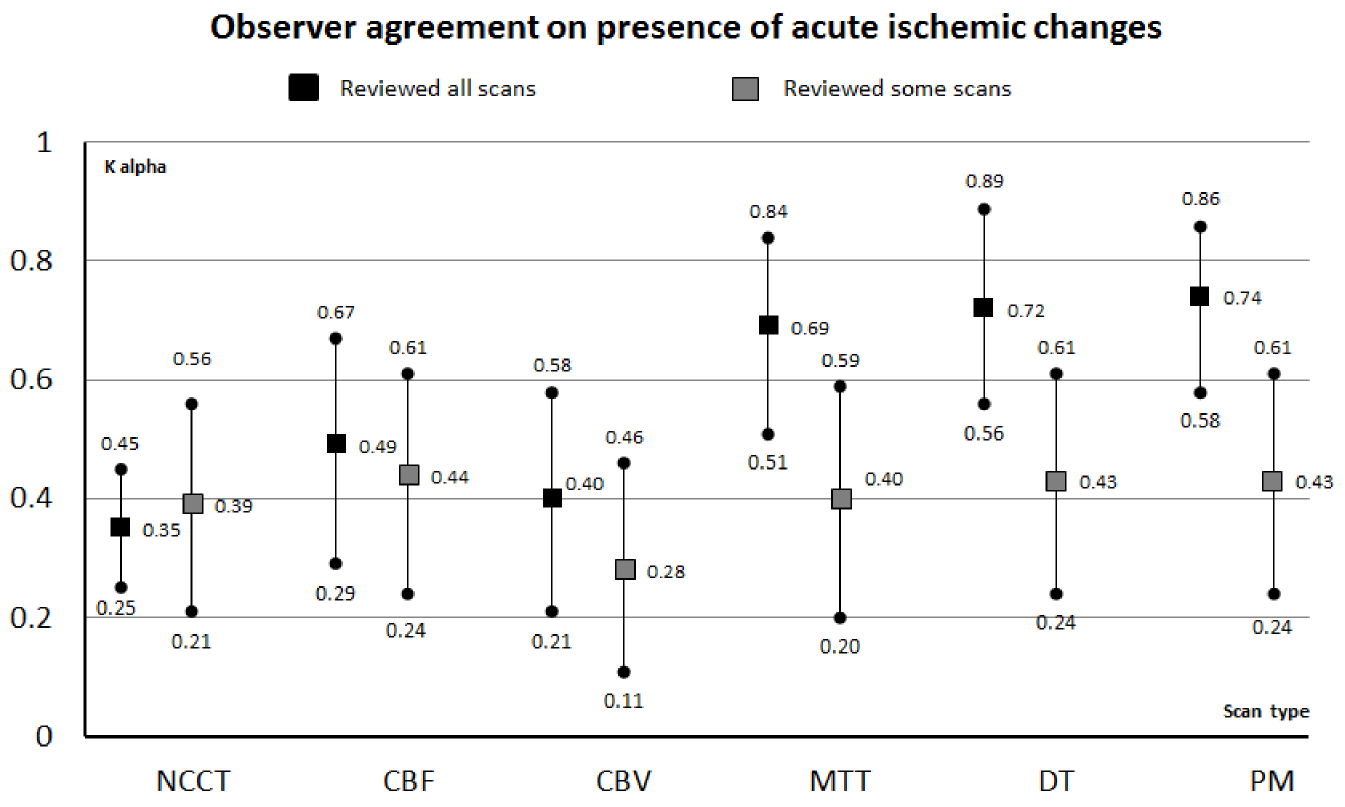

Supplementary figure II: Krippendorff alpha values and 95% confidence intervals for presence of acute ischemic changes in NCCT and perfusion maps (K. alpha= Krippendorff alpha, CI= confidence interval ,NCCT= non contrast computed topography, CBF= cerebral blood flow, CBV= cerebral blood volume, MTT= mean transient time, DT= delay time, PM= penumbra map)

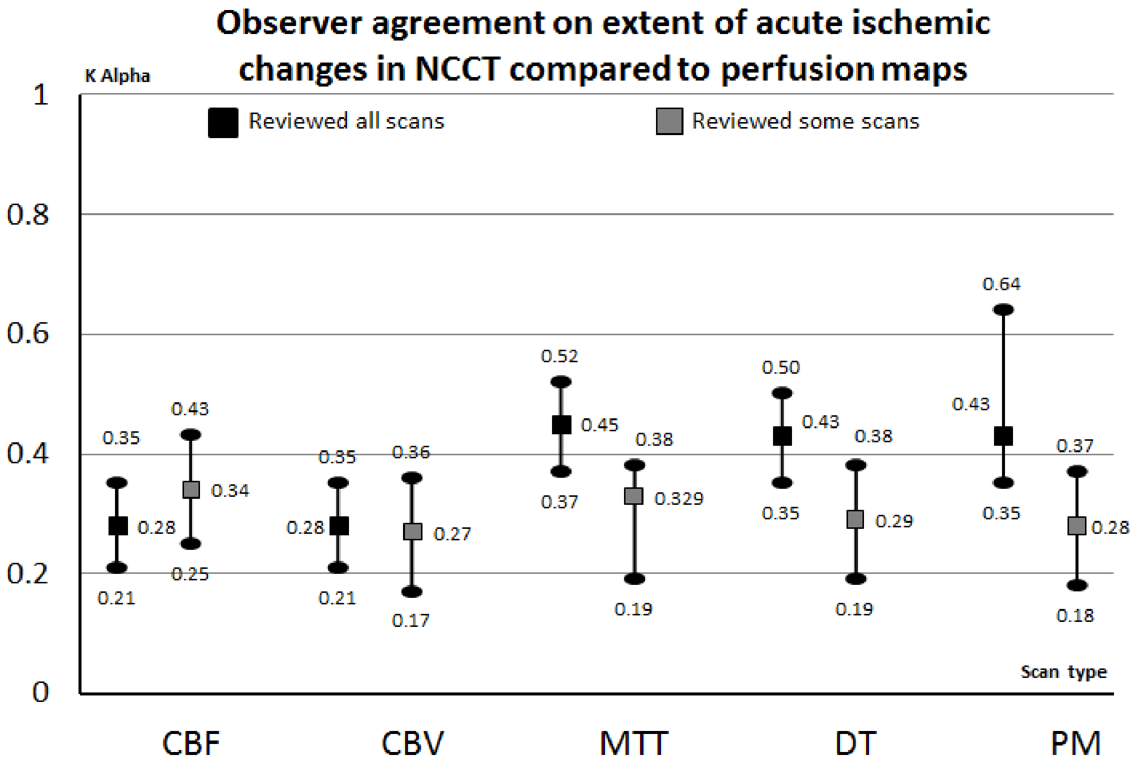

Supplementary figure III: Krippendorff alpha value and 95% confidence interval for observer agreement on extent of acute ischemic changes in NCCT compared to perfusion map (K. alpha= Krippendorff alpha, CI= confidence interval ,NCCT= non contrast computed topography, CBF= cerebral blood flow, CBV= cerebral blood volume, MTT= mean transient time, DT= delay time, PM= penumbra map)

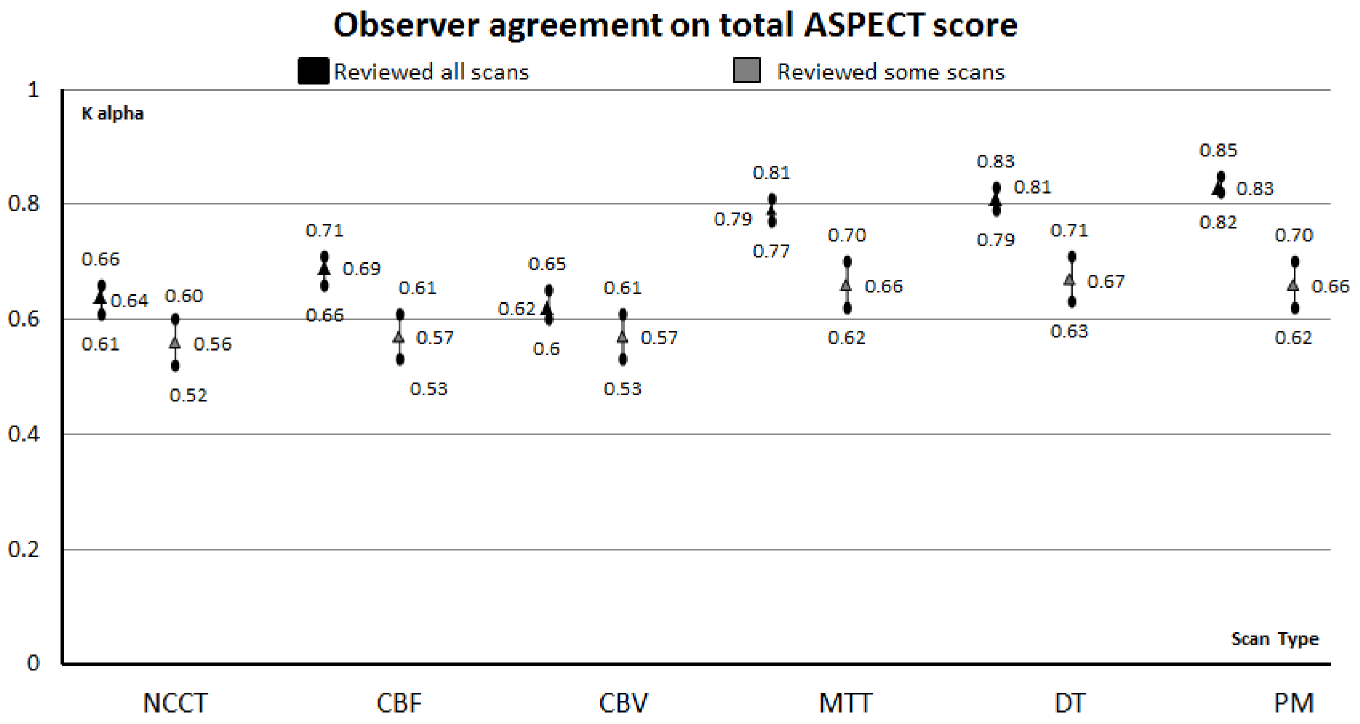

Supplementary figure IV: Krippendorff alpha values and 95% confidence intervals on presence for total ASPECT score in NCCT and perfusion maps (K. alpha= Krippendorff alpha, CI= confidence interval,NCCT= non contrast computed topography, CBF= cerebral blood flow, CBV= cerebral blood volume, MTT= mean transient time, DT= delay time, PM= penumbra map)Observer agreement on ASPECT score for NCCT and perfusion maps

Observer agreement on presence of acute ischemic changes in different observer specialities							
**K. alpha (CI)**		**NCCT**	**CBF**	**CBV**	**MTT**	**DT**	**PM**
Observer Speciality	Neuro-radiologist	0.49 (0.30-0.66)	0.62 (0.45-0.78)	0.49 (0.31-0.67)	0.79 (0.64-0.93)	0.79 0.64-0.94)	0.79 (0.61-0.91)
	Neurologist	0.32 (0.13-0.51)	0.32 (0.13-0.51)	0.29 (0.10-0.48)	0.60 (0.40-0.75)	0.61 (0.42-0.77)	0.64 (0.46-0.79)
	Stroke physician	0.51 (0.34-0.66)	0.48 (0.28-0.65)	0.27 (0.09-0.44)	0.49 (0.30-0.66)	0.52 (0.33-0.70)	0.52 (0.33-0.70)
	Other	0.32 (0.13-0.50)	0.28 (0.11-0.46)	0.40 (0.22-0.59)	0.48 (0.29-0.55)	0.44 (0.33-0.55)	0.43 (0.32-0.54)

Supplementary table IV: Krippendorff alpha values and 95% confidence intervals for presence of acute ischemic changes in NCCT and perfusion maps in observers of different specialities.(K. alpha= Krippendorff alpha, CI= confidence interval,NCCT= non contrast computed topography, CBF= cerebral blood flow, CBV= cerebral blood volume, MTT= mean transient time, DT= delay time, PM= penumbra map)

Observer agreement on presence of acute ischemic changes in observers with different experience in their specialities							
	**K. alpha (CI)**	**NCCT**	**CBF**	**CBV**	**MTT**	**DT**	**PM**
Years of specialisation	5 or less years	0.36 (0.17-0.55)	0.36 (0.18-0.53)	0.38 (0.18-0.56)	0.60 (0.41-0.77)	0.61 (0.43-0.79)	0.64 (0.45-0.82)
	6 to 15 years	0.36 (0.26-0.46)	0.55 (0.37-0.73)	0.36 (0.17-0.53)	0.66 (0.49-0.82)	0.70 (0.53-0.84)	0.70 (0.52-0.84)
	>15 years	0.50 (0.31-0.66)	0.61 (0.42-0.78)	0.38 (0.19-0.57)	0.63 (0.44-0.80)	0.66 (0.49-0.84)	0.66 (0.47-0.81)

Supplementary table V: Krippendorff alpha values and 95% confidence intervals for presence of acute ischemic changes in NCCT and perfusion maps in observers with different experience on their specialities.(K. alpha= Krippendorff alpha, CI= confidence interval ,NCCT= non contrast computed topography, CBF= cerebral blood flow, CBV= cerebral blood volume, MTT= mean transient time, DT= delay time, PM= penumbra map)

Observer agreement on presence of acute ischemic changes in observers with different experience of stroke imaging							
	**K. alpha (CI)**	**NCCT**	**CBF**	**CBV**	**MTT**	**DT**	**PM**
How often do you review stroke imaging?	Daily	0.43 (0.26-0.61)	0.51 (0.31-0.68)	0.38 (0.19-0.56)	0.65 (0.46-0.84)	0.68 (0.51-0.84)	0.70 (0.54-0.86)
	Less than daily	0.41 (0.22-0.58)	0.38 (0.19-0.55)	0.37 (0.18-0.54)	0.56 (0.36-0.73)	0.54 (0.35-0.71)	0.53 (0.34-0.71)

Supplementary table VI: Krippendorff alpha values and 95% confidence intervals for presence of acute ischemic changes in NCCT and perfusion maps in observers of experience with stroke imaging (K. alpha= Krippendorff alpha, CI= confidence interval,NCCT= non contrast computed topography, CBF= cerebral blood flow, CBV= cerebral blood volume, MTT= mean transient time, DT= delay time, PM= penumbra map)

Observer agreement on presence of acute ischemic changes in observers with different experience of perfusion imaging							
	**K. alpha (CI)**	**NCCT**	**CBF**	**CBV**	**MTT**	**DT**	**PM**
How often do you view perfusion imaging?	Weekly	0.47 (0.28-0.63)	0.53 (0.35-0.71)	0.37 (0.17-0.56)	0.63 (0.45-0.80)	0.67 (0.49-0.82)	0.67 (0.49-0.82)
	Monthly	0.33 (0.11-0.53)	0.54 (0.34-0.72)	0.32 (0.13-0.51)	0.67 (0.46-0.83)	0.70 (0.51-0.86)	0.74 (0.58-0.89)
	Less frequently	0.44 (0.26-0.60)	0.37 (0.19-0.54)	0.37 (0.18-0.54)	0.59 (0.41-0.75)	0.59 (0.41-0.77)	0.61 (0.42-0.79)

Supplementary table VII : Krippendorff alpha values and 95% confidence intervals for presence of acute ischemic changes in NCCT and perfusion maps in observers of different experience with perfusion imaging. (K. alpha= Krippendorff alpha, CI= confidence interval ,NCCT= non contrast computed topography, CBF= cerebral blood flow, CBV= cerebral blood volume, MTT= mean transient time, DT= delay time, PM= penumbra map)

Observer agreement on presence of acute ischemic changes in different scan subtypes							
**K. alpha (CI)**		**NCCT**	**CBF**	**CBV**	**MTT**	**DT**	**PM**
Perfusion deficit	Large	0.49 (0.27-0.69)	0.13 (0.45-0.20)	0.26 (0.04-0.47)	0.26 (0.15-0.60)	0.26 (-0.15-0.60)	0.26 (0.15-0.60)
	Small/medium	0.21 (0.02-0.42)	0.25 (0.04-0.46)	0.19 (-0.01-0.37)	0.26 (-0.06-0.57)	0.24 (-0.09-0.59)	0.24 (-0.11-0.58)
Perfusion z-axis	Less than 40 mm	0.33 (0.14-0.53)	0.34 (0.15-0.51)	0.25 (0.05-0.44)	0.42 (0.22-0.59)	0.43 (0.25-0.61)	0.43 (0.25-0.61)
	40 mm	0.41 (0.21-0.61)	0.51 (0.32-0.68)	0.41 (0.22-0.59)	0.75 (0.59-0.91)	0.79 (0.63-0.94)	0.83 (0.69-0.97)
	More than 40 mm	0.44 (0.26-0.62)	0.55 (0.35-0.74)	0.43 (0.25-0.59)	0.62 (0.44-0.80)	0.66 (0.48-0.83)	0.66 (0.48-0.86)

Supplementary table VIII: Krippendorff alpha values and 95% confidence intervals for presence of acute ischemic changes in NCCT and perfusion maps in different scan subtypes. (K. alpha= Krippendorff alpha, CI= confidence interval, NCCT= non contrast computed topography, CBF= cerebral blood flow, CBV= cerebral blood volume, MTT= mean transient time, DT= delay time, PM= penumbra map)

K. alpha (CI)		CBF	CBV	MTT	DT	PM
**Observer Speciality**	Neuro-radiologist	0.50(0.41-0.57)	0.42(0.33-0.51)	0.57(0.50-0.65)	0.56(0.48-0.63)	0.56(0.49-0.64)
	Neurologist	0.34(0.15-0.52)	0.31(0.22-0.40)	0.56(0.48-0.63)	0.51(0.43-0.59)	0.49(0.40-0.57)
	Stroke physician	0.35(0.25-0.44)	0.23(0.12-0.33)	0.34(0.24-0.44)	0.37(0.27-0.46)	0.36(0.26-0.46)
	Other	0.27(0.15-0.39)	0.36(0.29-0.66)	0.39(0.28-0.51)	0.44(0.33-0.55)	0.43(0.32-0.54)
**Years of specialisation**	5 or less years	0.37(0.28-0.46)	0.38(0.29-0.47)	0.49(0.41-0.58)	0.48(0.40-0.56)	0.51(0.43-0.59)
	6 to 15 years	0.44(0.35-0.53)	0.32(0.23-0.43)	0.57(0.49-0.65)	0.57(0.49-0.64)	0.54(0.46-0.62)
	>15 years	0.42(0.3-0.50)	0.30(0.21-0.41)	0.41(0.32-0.49)	0.42(0.33-0.50)	0.43(0.34-0.51)
**How often do you review stroke imaging?**	Daily	0.43(0.34-0.51)	0.36(0.26-0.45)	0.42(0.34-0.50)	0.51(0.43-0.59)	0.41(0.34-0.49)
	Less than daily	0.37(0.18-0.54)	0.40(0.31-0.48)	0.52(0.43-0.61)	0.48(0.36-0.59)	0.46(0.36-0.56)
**How often do you view perfusion imaging?**	Weekly	0.45(0.36-0.53)	0.35(0.25-0.44)	0.51(0.43-0.60)	0.52(0.43-0.60)	0.52(0.44-0.60)
	Monthly	0.53(0.46-0.61)	0.35(0.25-0.44)	0.56(0.48-0.63)	0.56(0.48-0.63)	0.58(0.51-0.65)
	Less frequently	0.33(0.23-0.42)	0.37(0.28-0.46)	0.47(0.39-0.56)	0.45(0.37-0.53)	0.46(0.37-0.54)

Supplementary table IX: Krippendorff alpha values and 95% confidence intervals for extent of acute ischemic changes in NCCT compared to different perfusion maps for different observer subtypes.(K. alpha= Krippendorff alpha, CI= confidence interval, NCCT= non contrast computed topography, CBF= cerebral blood flow, CBV= cerebral blood volume, MTT= mean transient time, DT= delay time, PM= penumbra map)

Observer agreement on extent of acute ischemic changes in NCCT compared to perfusion maps in different scan subgroups						
**K.alpha (CI)**		**CBF**	**CBV**	**MTT**	**DT**	**PM**
Perfusion deficit	Large	0.13(0.05-0.20)	0.14(0.08-0.21)	0.19(0.07-0.30)	0.19(0.08-0.30)	0.21(0.09-032)
	Small/medium	0.16(0.09-0.22)	0.11(0.04-0.18)	0.17(0.09-0.26)	0.13(0.05-0.22)	0.13(0.05-0.27)
Perfusion z-axis	Less than 40 mm	0.28(0.19-0.38)	0.28(0.19-0.38)	0.13(0.06-0.21)	0.31(0.22-0.39)	0.36(0.28-0.46)
	40 mm	0.48(0.40-0.56)	0.42(0.33-0.50)	0.60(0.53-0.67)	0.58(0.51-0.65	0.58(0.51-0.65)
	More than 40 mm	0.40(0.31-0.48)	0.38(0.29-0.47)	0.48(0.39-0.57)	0.50(0.41-0.58)	0.51(0.42-0.59)

Supplementary table X: Krippendorff alpha values and 95% confidence intervals for extent of acute ischemic changes in NCCT compared to different perfusion maps in different scan subtypes.(K. alpha= Krippendorff alpha, CI= confidence interval, NCCT= non contrast computed topography, CBF= cerebral blood flow, CBV= cerebral blood volume, MTT= mean transient time, DT= delay time, PM= penumbra map)

Observer agreement on total ASPECT score in observers of different specialities							
**K.alpha (CI)**		**NCCT**	**CBF**	**CBV**	**MTT**	**DT**	**PM**
Observer Speciality	Neuro-radiologist	0.66 (0.64-0.68)	0.78 (0.76-0.80)	0.68 (0.65-0.70)	0.83 (0.81-0.85)	0.87 (0.85-0.88)	0.89 (0.88-0.90)
	Neurologist	0.52 (0.48-0.57)	0.59 (0.54-0.63)	0.55 (0.50-0.59)	0.71 (0.67-0.74)	0.71 (0.67-0.75)	0.75 (0.72-0.78)
	Stroke physician	0.67 (0.62-0.71)	0.76 (0.73-0.80)	0.67 (0.62-0.72)	0.76 (0.71-0.80)	0.73 (0.67-0.78)	0.71 (0.65-0.76)
	Other	0.54 (0.42-0.64)	0.59 (0.49-0.69)	0.75 (0.67-0.2)	0.76 (0.70-0.82)	0.80 (0.75-0.86)	0.81 (0.76-0.86)

Supplementary table XI: Krippendorff alpha values and 95% confidence intervals total ASPECT score in NCCT and different perfusion maps for observers of different specialities (K. alpha= Krippendorff alpha, CI= confidence interval,NCCT= non-contrast computed topography, CBF= cerebral blood flow, CBV= cerebral blood volume, MTT= mean transient time, DT= delay time, PM= penumbra map)

Observer agreement on total ASPECT score in observers with different experience in their specialities							
**K. alpha (CI)**		**NCCT**	**CBF**	**CBV**	**MTT**	**DT**	**PM**
Years of specialisation	5 or less years	0.58 (0.57-0.61)	0.64 (0.62-0.67)	0.56 (0.53-0.59)	0.79 (0.77-0.81)	0.78 (0.76-0.80)	0.78 (0.77-0.80)
	6 to 15 years	0.64 (0.61-0.69)	0.73 (0.71-0.76)	0.67 (0.65-0.70)	0.82 (0.80-0.84)	0.83 (0.81-0.85)	0.83 (0.81-0.85)
	>15 years	0.59 (0.54-0.65)	0.76 (0.72-0.80)	0.60 (0.53-0.66)	0.65 (0.59-0.71)	0.77 (0.72-0.82)	0.79 (0.74-0.83)

Supplementary table XII: Krippendorff alpha values and 95% confidence intervals total ASPECT score in NCCT and different perfusion maps for observers of different experience in their specialities (K. alpha= Krippendorff alpha, CI= confidence interval,NCCT= non-contrast computed topography, CBF= cerebral blood flow, CBV= cerebral blood volume, MTT= mean transient time, DT= delay time, PM= penumbra map)

Observer agreement on total ASPECT score in observers with different experience with stroke imaging							
**K. alpha (CI)**		**K. alpha (CI)**	**K. alpha (CI)**	**K. alpha (CI)**	**K. alpha (CI)**	**K. alpha (CI)**	**K. alpha (CI)**
How often do you review stroke imaging?	Daily	0.66 (0.64-0.68)	0.75 (0.73-0.77)	0.66 (0.64-0.68)	0.79 (0.77-0.81)	0.81 (0.81-0.84)	0.83 (0.81-0.84)
	Less than daily	0.51 (0.45-0.56)	0.48 (0.42-0.53)	0.54 (0.49-0.59)	0.71 (0.67-0.75)	0.69 (0.65-0.73)	0.72 (0.68-0.75)

Supplementary table XIII: Krippendorff alpha values and 95% confidence intervals total ASPECT score in NCCT and different perfusion maps for observers with different experience with stroke imaging (K. alpha= Krippendorff alpha, CI= confidence interval ,NCCT= non-contrast computed topography, CBF= cerebral blood flow, CBV= cerebral blood volume, MTT= mean transient time, DT= delay time, PM= penumbra map)

Observer agreement on total ASPECT score in observers with different experience with perfusion imaging							
**K. alpha (CI)**		**NCCT**	**CBF**	**CBV**	**MTT**	**DT**	**PM**
How often do you view perfusion imaging?	Weekly	0.63 (0.60-0.65)	0.74 (0.72-0.76)	0.62 (0.60-0.65)	0.77 (0.74-0.79)	0.79 (0.77-0.81)	0.79 (0.77-0.82)
	Monthly	0.60 (0.54-0.65)	0.60 (0.54-0.65)	0.66 (0.61-0.71)	0.83 (0.80-0.86)	0.84 (0.80-0.87)	0.84 (0.81-0.87)
	Less frequently	0.60 (0.58-0.63)	0.58 (0.55-0.61)	0.61 (0.58-0.63)	0.75 (0.73-0.77)	0.61 (0.58-0.63)	0.80 (0.78-0.82)

Supplementary table XIV: Krippendorff alpha values and 95% confidence intervals total ASPECT score in NCCT and different perfusion maps for observers with different experience with perfusion imaging (K. alpha= Krippendorff alpha, CI= confidence interval NCCT= non-contrast computed topography, CBF= cerebral blood flow, CBV= cerebral blood volume, MTT= mean transient time, DT= delay time, PM= penumbra map)

Observer agreement on total ASPECT score in different scan subtypes							
**K. alpha (CI)**		**NCCT**	**CBF**	**CBV**	**MTT**	**DT**	**PM**
Perfusion deficit	Large	0.54 (0.51-0.56)	0.51 (0.48-0.54)	0.59 (0.56-0.61)	0.39 (0.63-0.43)	0.40 (0.36-0.43)	0.39 (0.36-0.43)
	Small/medium	0.26 (0.22-0.30)	0.31 (0.27-0.35)	0.25 (0.20-0.30)	0.49 (0.45-0.52)	0.51 (0.47-0.55)	0.56 (0.53-0.60)
Perfusion z-axis	Less than 40 mm	0.41 (0.37-0.45)	0.46 (0.41-0.51)	0.41 (0.35-0.48)	0.50 (0.46-0.54)	0.47 (0.42-0.53)	0.50 (0.45-0.53)
	40 mm	0.64 (0.62-0.67)	0.68 (0.66-0.71)	0.57 (0.55-0.60)	0.78 (0.76-0.80)	0.80 (0.78-0.82)	0.84 (0.83-0.85)
	More than 40 mm	0.60 (0.58-0.63)	0.70 (0.68-0.72)	0.76 (0.74-0.78)	0.73 (0.70-0.75)	0.75 (0.73-0.77)	0.72 (0.69-0.74)

Supplementary table XV: Krippendorff alpha values and 95% confidence intervals total ASPECT score in NCCT and different perfusion maps in different scan subtypes (K. alpha= Krippendorff alpha, CI= Confidence ,NCCT= non-contrast computed topography, CBF= cerebral blood flow, CBV= cerebral blood volume, MTT= mean transient time, DT= delay time, PM= penumbra map)

Intra-observer agreement presence of acute ischemic changes in observers of different specialities							
	**Mean K. alpha (CI of Mean)**	**NCCT**	**CBF**	**CBV**	**MTT**	**DT**	**PM**
Observer Speciality	Neuro-radiologist	0.49 (0.14-0.83)	0.73 (0.47-0.98)	0.49 (0.28-0.69)	0.80 (0.44- 1.15)	0.80 (0.44-1.15)	0.80 (0.44-1.15)
	Neurologist	0.81 (0.58-1.03)	0.59 (0.27-0.90)	0.26 (-0.08-0.60)	0.77 (0.34-1.19)	0.93 (0.76-1.11)	0.93 (0.76-1.10)
	Stroke physician	0.50 (-1.91-2.91)	0.39 (-7.36-8.14)	0.08 (-1.86-1.70)	0.39 (-7.36-8.14)	0.39 (-7.36-8.14)	0.39 (-7.36-8.14)
	Other	0.45 (-1.32-2.23)	0.19 (-1.40-1.78)	0.70 (-3.18-1.70)	0.85 (-1.12-2.81)	0.85 (-1.12-2.81)	0.85 (-1.12-2.81)
ANOVA	t	0.26	0.30	0.06	0.68	0.40	0.40

Supplementary table XVI: Intra-observer Krippendorff alpha mean values and 95% confidence intervals of the mean for presence of acute ischemic changes in NCCT and different perfusion maps in observers of different specialities (K. alpha= Krippendorff alpha, CI= confidence interval,NCCT= non-contrast computed topography, CBF= cerebral blood flow, CBV= cerebral blood volume, MTT= mean transient time, DT= delay time, PM= penumbra map).

Intra Observer agreement presence of acute ischemic changes in observers of different experience in their specialities							
	**Mean K. alpha (CI of Mean)**	**NCCT**	**CBF**	**CBV**	**MTT**	**DT**	**PM**
Years of specialisation	5 or less years	0.57 (0.28-0.85)	0.54 (0.19-0.89)	0.44 (0.14-0.75)	0.66 (0.29-1.03)	0.79 (0.08-1.43)	0.79 (0.49-1.08)
	6 to 15 years	0.73 (0.19-0.91)	0.72 (0.41-1.02)	0.28 (-0.19-0.75)	0.90 (0.57-1.22)	0.89 (0.57-1.22)	0.90 (0.57-1.23)
	>15 years	0.54 (0.17-0.91)	0.51 (-0.42-1.06)	0.30 (-0.17-0.77)	0.75 (0.08-1.43	0.76 (0.08-1.43)	0.76 (0.08-1.43)
ANOVA	t	0.067	0.70	0.68	0.68	0.86	0.86

Supplementary table XVII: Intra-observer Krippendorff alpha mean values and 95% confidence intervals of the mean for presence of acute ischemic changes in NCCT and different perfusion maps in observers of different experience in their specialities (K. alpha= Krippendorff alpha, CI= confidence interval ,NCCT= non-contrast computed topography, CBF= cerebral blood flow, CBV= cerebral blood volume, MTT= mean transient time, DT= delay time, PM= penumbra map

Intra Observer agreement Presence of acute ischemic changes in observers of different experience with stroke imaging							
	**Mean K. alpha (CI of Mean)**	**NCCT**	**CBF**	**CBV**	**MTT**	**DT**	**PM**
How often do you review stroke imaging?	Daily	0.62 (0.43-0.80)	0.60 (0.39-0.81)	0.34 (0.15-0.53)	0.73 (0.49-0.98)	0.80 (0.58-1.01)	0.80 (0.58-1.02)
	Less than daily	0.45 (-1.33-2.29)	0.38 (-3.63-4.38)	0.54 (-1.37-2.45)	0.85 (-1.12-2.81)	0.85 (-1.12-2.81)	0.45 (-1.12-2.81)
ANOVA	t	0.50	0.44	0.45	0.73	0.87	0.87

Supplementary table XVIII: Intra-observer Krippendorff alpha mean values and 95% confidence intervals of the mean for presence of acute ischemic changes in NCCT and different perfusion maps in observers of different experience in stroke imaging (K. alpha= Krippendorff alpha, CI= confidence interval, NCCT= non-contrast computed topography, CBF= cerebral blood flow, CBV= cerebral blood volume, MTT= mean transient time, DT= delay time, PM= penumbra map)

Intra Observer agreement Presence of acute ischemic changes in observers of different experience with perfusion imaging							
	**Mean K. alpha (CI of Mean)**	**NCCT**	**CBF**	**CBV**	**MTT**	**DT**	**PM**
How often do you view perfusion imaging?	Weekly	0.50 (0.07-0.93)	0.75 (0.44-1.06)	0.40 (0.10-0.69)	0.77 (0.34-1.19)	0.77 (0.34-1.09)	0.78 (0.36-1.20)
	Monthly	0.75 (0.26-1.23)	0.62 (0.17-1.07)	0.48 (-0.28-1.23)	0.65 (-0.11-1.40)	0.90 (0.57-1.22)	0.77 (0.34-1.19)
	Less frequently	0.59 (0.37-0.81)	0.40 (-0.01-0.80)	0.27 (-0.04-0.58)	0.78 (0.36-1.20)	0.78 (0.36-1.20)	0.78 (0.36-1.20)
ANOVA	t	0.52	0.24	0.64	0.88	0.86	0.86

Supplementary table XIX: Intra-observer Krippendorff alpha mean values and 95% confidence intervals of the mean for presence of acute ischemic changes in NCCT and different perfusion maps in observers of different experience in perfusion imaging (K. alpha= Krippendorff alpha, CI= confidence interval ,NCCT= non-contrast computed topography, CBF= cerebral blood flow, CBV= cerebral blood volume, MTT= mean transient time, DT= delay time, PM= penumbra map)

Intra-observer agreement on extent of acute ischemic changes in NCCT compared to perfusion maps in observers of different specialities						
**Mean K. alpha (CI of Mean)**		**CBF**	**CBV**	**MTT**	**DT**	**PM**
Observer Speciality	Neuro-radiologist	0.45 (0.14-0.76)	0.34 (0.14-0.54)	0.67 (0.46-0.87)	0.70 (0.50-0.90)	0.68 (0.66-0.89)
	Neurologist	0.39 (0.02-0.75)	0.28 (-0.08-0.64)	0.79 (0.45-1.13)	0.77 (0.57-0.96)	0.74 (0.51-0.97)
	Stroke physician	0.42 (-6.76-7.59)	-0.16 (-7.08-6.77)	0.38 (-7.24-8.00)	0.25 (-5.66-6.15)	0.68 (0.46-0.89)
	Other	0.51 (-2.80-3.81)	0.60 (-2.70-3.90)	0.79 (-1.69-3.26)	0.69 (-0.58-1.96)	0.56 (0.18-0.94)
ANOVA	t	0.98	0.22	0.55	0.16	0.21

Supplementary table XX: Intra-observer Krippendorff alpha mean values and 95% confidence intervals of the mean for difference between acute ischemic changes in NCCT and different perfusion maps in observers of different specialities (K. alpha= Krippendorff alpha, CI= confidence interval NCCT= non-contrast computed topography, CBF= cerebral blood flow, CBV= cerebral blood volume, MTT= mean transient time, DT= delay time, PM= penumbra map)

Intra-observer agreement on extent of acute ischemic changes in NCCT compared to perfusion maps in observers of different experience in their specialities						
	**Mean K. alpha (CI of Mean)**	**CBF**	**CBV**	**MTT**	**DT**	**PM**
Years of specialisation	5 or less years	0.48 (0.15-0.80)	0.39 (0.14-0.65)	0.73 (0.48-0.98)	0.72 (0.57-0.88)	0.69 (0.53-0.85)
	6 to 15 years	0.67 (0.18-1.15)	0.33 (-0.22-0.89)	0.89 (0.62-1.17)	0.76 (0.52-1.00)	0.72 (0.38-1.06)
	>15 years	0.16 (-0.06-0.39)	0.10 (-0.49-0.69)	0.46 (-0.08-0.99)	0.51 (-0.05-1.08)	0.48 (-0.08-1.03)
ANOVA	t	0.09	0.39	0.15	0.36	0.38

Supplementary table XXI: Intra-observer Krippendorff alpha mean values and 95% confidence intervals of the mean for difference between acute ischemic changes in NCCT and different perfusion maps in observers of different experience in their specialities (K. alpha= Krippendorff alpha, CI= confidence interval NCCT= non-contrast computed topography, CBF= cerebral blood flow, CBV= cerebral blood volume, MTT= mean transient time, DT= delay time, PM= penumbra map).

Intra-observer agreement on extent of acute ischemic changes in NCCT compared to perfusion maps in observers of different experience with stroke imaging						
	**Mean K. alpha (CI of Mean)**	**CBF**	**CBV**	**MTT**	**DT**	**PM**
How often do you review stroke imaging?	Daily	0.42 (0.21-0.62)	0.26 (0.05-0.46)	0.68 (0.48-0.88)	0.66 (0.49-0.82)	0.61 (0.45-0.78)
	Less than daily	0.52 (-2.91-3.95)	0.57 (-2.29-3.42)	0.79 (-1.70-3.26)	0.79 (-1.75-3.33)	0.79 (-1.75-3.33)
ANOVA	t	0.72	0.28	0.69	0.55	0.45

Supplementary table XXII: Intra-observer Krippendorff alpha mean values and 95% confidence intervals of the mean for difference between acute ischemic changes in NCCT and different perfusion maps in observers of different experience with stroke imaging (K. alpha= Krippendorff alpha, CI= confidence interval NCCT= non-contrast computed topography, CBF= cerebral blood flow, CBV= cerebral blood volume, MTT= mean transient time, DT= delay time, PM= penumbra map).

Intra-observer agreement on extent of acute ischemic changes in NCCT compared to perfusion maps in observers of different experience with perfusion imaging						
	**Mean K. alpha (CI of Mean)**	**CBF**	**CBV**	**MTT**	**DT**	**PM**
How often do you view perfusion imaging?	Weekly	0.59 (0.18-1.01)	0.38 (-0.17-0.60)	0.84 (0.63-1.07)	0.76 (0.56-0.95)	0.76 (0.56-0.95)
	Monthly	0.40 (-0.23-1.02)	0.32 (-0.33-0.96)	0.58 (0.06-1.11)	0.68 (0.49-0.87)	0.57 (0.37-0.78)
	Less frequently	0.30 (0.02-0.60)	0.20 (-0.24-0.64)	0.62 (0.22-1.02)	0.59 (0.21-0.98)	0.57 (0.18-0.95)
ANOVA	t	0.38	0.69	0.40	0.62	0.48

Supplementary table XXIII: Intra-observer Krippendorff alpha mean values and 95% confidence intervals of the mean for difference between acute ischemic changes in NCCT and different perfusion maps in observers of different experience with perfusion imaging (K. alpha= Krippendorff alpha, CI= confidence interval NCCT= non-contrast computed topography, CBF= cerebral blood flow, CBV= cerebral blood volume, MTT= mean transient time, DT= delay time, PM= penumbra map)

Intra-observer agreement on total ASPECT score in observers of different specialities							
	**Mean K. alpha (CI of Mean)**	**NCCT**	**CBF**	**CBV**	**MTT**	**DT**	**PM**
Observer Speciality	Neuro-radiologist	0.62 (0.46-0.78)	0.55 (0.31-0.79)	0.57 (0.24-0.91)	0.83 (0.73-0.93)	0.79 (0.67-0.91)	0.77 (0.64-0.90)
	Neurologist	0.63 (0.23-1.03)	0.55 (0.09-1.00)	0.47 (-0.04-0.94)	0.66 (0.22-1.10)	0.88 (0.53-0.99)	0.83 (0.67-0.99)
	Stroke physician	0.51 (-0.19-1.20)	0.55 (0.35-0.74)	0.35 (-1.75-2.44)	0.77 (-2.22-3.75)	0.79 (-1.94-3.52)	0.70 (-3.18-4.57)
	Other	0.55 (-2.25-3.35)	0.91 (-0.30-2.11)	0.45 (-5.21-6.10)	0.81 (-0.21-1.82)	0.73 (0.66-0.79)	0.85 (-0.62-2.30)
ANOVA	t	0.94	0.56	0.90	0.76	0.97	0.78

Supplementary table XXIV: Intra-observer Krippendorff alpha mean values and 95% confidence intervals of the mean for total ASPECT score in NCCT and different perfusion maps in observers of different specialities (K. alpha= Krippendorff alpha, CI= confidence interval NCCT= non-contrast computed topography, CBF= cerebral blood flow, CBV= cerebral blood volume, MTT= mean transient time, DT= delay time, PM= penumbra map)

Intra-observer agreement on total ASPECT score in observers of different experiences in their respective specialities							
	**Mean K. alpha (CI of Mean)**	**NCCT**	**CBF**	**CBV**	**MTT**	**DT**	**PM**
Years of specialisation	5 or less years	0.69 (0.25-1.14)	0.62 (0.32-0.93)	0.40 (0.01-0.80)	0.69 (0.40-0.98)	0.73 (0.57-0.88)	0.74 (0.62-0.87)
	6 to 15 years	0.54 (0.38-0.69)	0.69 (0.18-1.19)	0.58 (-0.06-0.57)	0.82 (0.51-1.13)	0.87 (0.65-1.09)	0.96 (0.87-1.05)
	>15 years	0.63 (0.32-0.93)	0.46 (0.17-0.75)	0.55 (0.23-0.87)	0.82 (0.60-1.04)	0.77 (0.60-0.94)	0.74 (0.48-0.81)
ANOVA	t	0.68	0.56	0.72	0.64	0.38	0.08

Supplementary table XXV: Intra-observer Krippendorff alpha mean values and 95% confidence intervals of the mean for total ASPECT score in NCCT and different perfusion maps in observers of different experience in their specialities (K. alpha= Krippendorff alpha, CI= confidence interval NCCT= non-contrast computed topography, CBF= cerebral blood flow, CBV= cerebral blood volume, MTT= mean transient time, DT= delay time, PM= penumbra map).

Intra-observer agreement on total ASPECT score in observers of different experiences of stroke imaging							
	**Mean K. alpha (CI of Mean)**	**NCCT**	**CBF**	**CBV**	**MTT**	**DT**	**PM**
How often do you review stroke imaging?	Daily	0.60 (0.45-0.75)	0.54 (0.37-0.70)	0.49 (0.28-0.69)	0.77 (0.61-0.93)	0.78 (0.68-0.88)	0.78 (0.68-0.88)
	Less than daily	0.61 (-1.42-2.64)	0.93 (0.93-1.06)	0.50 (-5.79-6.78)	0.70 (-0.32-1.08)	0.73 (0.66-0.79)	0.86 (-0.76-2.44)
ANOVA	t	0.96	0.05	0.98	0.75	0.68	0.60

Supplementary table XXVI: Intra-observer Krippendorff alpha mean values and 95% confidence intervals of the mean for total ASPECT score in NCCT and different perfusion maps in observers of different experience in stroke imaging (K. alpha= Krippendorff alpha, CI= confidence interval ,NCCT= non-contrast computed topography, CBF= cerebral blood flow, CBV= cerebral blood volume, MTT= mean transient time, DT= delay time, PM= penumbra map)

Intra-observer agreement on total ASPECT score in observers of different experiences of perfusion imaging							
	**Mean K. alpha (CI of Mean)**	**NCCT**	**CBF**	**CBV**	**MTT**	**DT**	**PM**
How often do you view perfusion imaging?	Weekly	0.67 (0.43-0.90)	0.64 (0.39-0.89)	0.57 (0.16-0.97)	0.79 (0.64-0.95)	0.82 (0.66-0.98)	0.81 (0.63-0.99)
	Monthly	0.48 (-0.21-1.16)	0.38 (-0.82-0.84)	0.44 (-0.30-1.19)	0.66 (-0.19-1.51)	0.88 (0.70-1.05)	0.91 (0.75-1.06)
	Less frequently	0.62 (0.48-0.76)	0.67 (0.32-1.01)	0.45 (0.07-0.82)	0.79 (0.64-0.94)	0.67 (0.52-0.82)	0.71 (0.54-0.89)
ANOVA	t	0.54	0.33	0.85	0.73	0.09	0.22

Supplementary table XXVII: Intra-observer Krippendorff alpha mean values and 95% confidence intervals of the mean for total ASPECT score in NCCT and different perfusion maps in observers of different experience in perfusion imaging (K. alpha= Krippendorff alpha, CI= confidence interval, NCCT= non-contrast computed topography, CBF= cerebral blood flow, CBV= cerebral blood volume, MTT= mean transient time, DT= delay time, PM= penumbra map)

References

1. Aviv RI, Mandelcorn J, Chakraborty S, Gladstone D, Malham S, Tomlinson G, et al. Alberta stroke program early ct scoring of ct perfusion in early stroke visualization and assessment. *AJNR. American journal of neuroradiology*. 2007;28:1975-1980
2. Popiela T, Pera J, Chrzan R, Wloch D, Urbanik A, Slowik A. Interobserver agreement in perfusion computed tomography evaluation in acute ischaemic stroke. *Neurologia i neurochirurgia polska*. 2008;42:391-395
3. Finlayson O, John V, Yeung R, Dowlatshahi D, Howard P, Zhang L, et al. Interobserver agreement of aspect score distribution for noncontrast ct, ct angiography, and ct perfusion in acute stroke. *Stroke; a journal of cerebral circulation*. 2013;44:234-236
4. van Seeters T, Biessels GJ, Niesten JM, van der Schaaf IC, Dankbaar JW, Horsch AD, et al. Reliability of visual assessment of non-contrast ct, ct angiography source images and ct perfusion in patients with suspected ischemic stroke. *PloS one*. 2013;8:e75615
5. Psychogios MN, Schramm P, Frolich AM, Kallenberg K, Wasser K, Reinhardt L, et al. Alberta stroke program early ct scale evaluation of multimodal computed tomography in predicting clinical outcomes of stroke patients treated with aspiration thrombectomy. *Stroke*. 2013;44:2188-2193
6. Khaw AV, Angermaier A, Michel P, Kirsch M, Kessler C, Langner S. Inter-rater agreement in three perfusion-computed tomography evaluation methods before endovascular therapy for acute ischemic stroke. *Journal of stroke and cerebrovascular diseases : the official journal of National Stroke Association*. 2016;25:960-968
7. Shankar JJ, Langlands G, Doucette S, Phillips S. Ct perfusion in acute stroke predicts final infarct volume- inter-observer study. *The Canadian journal of neurological sciences. Le journal canadien des sciences neurologiques*. 2016;43:93-97
8. Naylor J, Churilov L, Chen Z, Koome M, Rane N, Campbell BCV. Reliability, reproducibility and prognostic accuracy of the alberta stroke program early ct score on ct perfusion and non-contrast ct in hyperacute stroke. *Cerebrovascular diseases (Basel, Switzerland)*. 2017;44:195-202
